# PD112 Calcitonin Gene-Related Peptide Monoclonal Antibodies Reimbursed Under A Managed Access Protocol In Ireland

**DOI:** 10.1017/S0266462324003544

**Published:** 2025-01-07

**Authors:** Karen Finnigan, Amelia Smith, Claire Gorry, Sarah Clarke, Michael Barry

## Abstract

**Introduction:**

The calcitonin gene-related peptide monoclonal antibodies (CGRP MABs) erenumab, fremanezumab, and galcanezumab are reimbursed in Ireland under the High Tech Arrangement, subject to a managed access protocol (MAP), for the prophylaxis of chronic migraine in adults in whom three or more prophylactic treatments have failed. This study provides an overview of submitted reimbursement applications and the utilization of CGRP MABs.

**Methods:**

The MAP for CGRP MABs was introduced on 1 September 2021 and is operated by the Health Service Executive (HSE) Medicines Management Programme. Individual patient reimbursement applications for CGRP MABs submitted through an online reimbursement application system between 1 September 2021 and 30 April 2023 were reviewed. Utilization data from 1 September 2021 to 30 April 2023 were extracted from the HSE Primary Care Reimbursement Service national pharmacy reimbursement claims database for the High Tech Arrangement. Analysis was performed using SAS® 9.4 software.

**Results:**

A total of 1,517 reimbursement applications were submitted in the study period. Reimbursement was approved for 96.1 percent (n=1,458) of the applications. A total of 1,399 individual patients (mean age 45 years) were dispensed a CGRP MAB under the High Tech Arrangement between September 2021 and April 2023, the majority of whom were women (n=1,141). Almost 90 percent of patients were considered treatment adherent. In April 2023, the market share of the individual CGRP MABs on the High Tech Arrangement was 56 percent (n=599) for fremanezumab, 38.3 percent (n=409) for erenumab, and 5.7 percent (n=61) for galcanezumab.

**Conclusions:**

MAPs are part of the health technology management approach to drug reimbursement in the Irish healthcare setting, ensuring that reimbursement is in line with approved subgroups of the licensed indication. Used in conjunction with health technology assessment, MAPs enable access to high-cost drug treatments for patients with the greatest unmet need, while providing budgetary oversight and certainty for the payer.

